# Clinical Evaluation of Keratinized Tissue Gain Using a Free Gingival Graft Combined With Frenotomy in Patients With Aberrant Mandibular Frenal Attachment: A Three-Month Follow-Up Case Series

**DOI:** 10.7759/cureus.93724

**Published:** 2025-10-02

**Authors:** Mohammed Saleh, Nada Zazou, Manal Hosny

**Affiliations:** 1 Department of Periodontology and Oral Medicine, October University for Modern Sciences and Arts, Giza, EGY; 2 Department of Periodontology and Oral Medicine, Faculty of Dentistry, Cairo University, Giza, EGY

**Keywords:** free gingival graft, frenotomy, frenum, mucosal scar index, relapse

## Abstract

Introduction: The aim of the present study was to evaluate the treatment of aberrant frenum attachment using frenotomy combined with free gingival grafts (FGGs) with respect to the gain in the width of keratinized gingiva, relapse, mucosal healing, and postoperative pain, as well as scar formation.

Methodology: Twelve adult patients (three males and nine females) with aberrant mandibular frenum attachment underwent frenotomy combined with free gingival grafting at the Faculty of Dentistry, Cairo University, and October University for Modern Sciences and Arts. Frenotomy was performed using split-thickness incisions on both sides of the frenum, followed by apical relocation of the muscle through blunt dissection. A palatal FGG was then harvested and secured to the recipient site with resorbable sutures.

Results: Keratinized tissue increased significantly from 2.08 ± 0.34 mm to 8.83 ± 0.53 mm at three months (p < 0.001). The postoperative pain scores dropped significantly from 5.83 ± 0.35 on day 1 to 0.33 ± 0.14 on day 7 (p < 0.001). The "inflammatory proliferative remodelling" index showed successful healing with scores of 5.33 ± 0.26 (inflammatory), 4.00 ± 0.21 (proliferative), and 2.17 ± 0.11 (remodelling), with a total of 11.5 ± 0.45, indicating excellent healing in 83.3% of cases (p = 0.021). At three months, the frenum was located at a more apical level than baseline by 4.3 ± 0.43 mm, with a significant difference between its baseline and three-month location (p < 0.001). The mean scar index at three months was 4.17 ± 0.94, reflecting mild to moderate scarring.

Conclusion: This combined approach is an effective, safe, and predictable modality for treating aberrant labial mandibular frenum attachments in adults, particularly where periodontal health and soft tissue augmentation are the primary goals.

## Introduction

The term "mucogingival surgery" refers to any periodontal surgical procedure intended to preserve attached gingiva, eliminate frenulum, or deepen the vestibule, including techniques that intend to repair irregularities in the shape, location, and/or quantity of gingiva that surrounds teeth [1].

The frenum is a small anatomical structure or fold of mucous membrane containing muscle fibers that is found on the vestibular mucosa of both the maxilla and mandible. In the maxilla, it is most commonly found in the midline opposite to the central incisors and sometimes in the premolar region; however, in the mandible, it is present labial or lingual to the lower centrals connected to the body of the tongue [2].

Attention to the labial frenum has gained clinical importance, as it could be associated with unfavorable conditions, such as difficulty in maintaining proper oral hygiene with the accumulation of plaque, and could play a role in diastema formation and orthodontic treatment relapse. Moreover, it could have a direct pull effect on the gingival margin, leading to gingival recession [2]. In the presence of shallow vestibular depth and a narrow zone of keratinized gingiva, the mandibular frenum is regarded as abnormal. In these cases, surgical removal of the frenum is advised [3]. Several classifications of frena have been proposed. Mirko et al. described four types of labial frenum according to their insertion level: mucosal, gingival, papillary, and papilla-penetrating [3]. Generally, frena with insertions distant from the gingival margin are considered physiologic, whereas those inserting close to the gingival margin or interdental papilla are deemed abnormal or aberrant [3,4].

Although various surgical techniques exist for managing aberrant frena, such as frenectomy, V-Y plasty, and Z-plasty [5], these methods have limitations, including increased trauma, delayed healing, higher risk of relapse, and esthetic concerns due to scarring [2,6,7]. Frenotomy, by contrast, is a more conservative technique that repositions rather than completely removes the frenum, thereby reducing tissue trauma and postoperative discomfort [7,8]. However, frenotomy alone may not provide adequate resistance to muscle pull or ensure long-term stability, especially in patients with a narrow keratinized gingiva or shallow vestibule. The addition of a free gingival graft (FGG), considered the gold standard for augmenting keratinized tissue [9], enhances periodontal stability by creating a wider protective zone of attached gingiva and reducing the likelihood of recurrence. Therefore, the combination of frenotomy with FGG was chosen in this study, as it unites the benefits of functional release with soft tissue augmentation, aiming to minimize relapse, improve healing, and reduce scar formation.

Previous studies have described frenectomy with or without autogenous grafts [5-7,9] or the use of FGGs to augment keratinized tissue in non-frenum cases [10,11]. However, none have discussed all precise details of the frenotomy procedure and the use of an FGG in combination with the frenotomy procedure to increase the zone of keratinized tissue; thus, the objective of this case series is to evaluate the effectiveness of using this dual approach in terms of the gain in the width of keratinized gingiva, relapse, mucosal healing, and postoperative pain, as well as scar formation.

## Materials and methods

Study design

This case series included 12 adult patients (n = 12) with aberrant mandibular frenum attachment who underwent frenotomy combined with free gingival grafting, treated between October 2023 and August 2024 at the Faculty of Dentistry, Cairo University, and October University for Modern Sciences and Arts (MSA). It was registered in the U.S. National Institutes of Health Clinical Trials Registry and ClinicalTrials.gov, with identifier NCT06102642. The study was reviewed and approved by the Ethics Committee of Scientific Research, Faculty of Dentistry, Cairo University, with registration code 28-5-23.

Study outcomes

Primary Outcome

The primary outcome was the width of keratinized tissue (WKT), measured in mm using a UNC periodontal probe at baseline (14 days), two months, and three months postoperatively. The final gain of keratinized tissue was calculated as the difference between baseline and three-month follow-up.

Secondary Outcomes

Wound healing: Mucosal healing was assessed using the Inflammatory-Proliferative-Remodeling (IPR) index as described by Hamzani and Chaushu (2018) [12]. It was assessed at five days, 14 days, and six weeks postoperatively. This scale evaluates three healing phases, namely, inflammatory, proliferative, and remodelling, using defined clinical parameters, with a total score ranging from 0 to 16. Scores of 11-16 indicate excellent healing, 5-10 acceptable healing, and 0-4 poor healing. 

Postoperative pain: It is evaluated using the Numerical Rating Scale (NRS) [13], where patients rated pain from 0 (no pain) to 10 (worst imaginable pain). Pain scores were collected on the seventh postoperative day.

Relapse: It is defined as the reappearance or reattachment of the frenum [14]. It was evaluated by measuring the distance between the most coronal point of frenum attachment and the incisal edge of the mandibular central incisors at the site corresponding to the frenum. Measurements were performed using a calibrated periodontal probe at baseline (preoperative) and at 3 months postoperatively, with the incisal edge of the mandibular central incisors serving as a fixed reference point to standardize probe positioning. A reduction in this distance indicated coronal migration or relapse of the frenum, whereas maintenance or an increase reflected stable apical repositioning.

Scar formation: It was assessed at three months using the Mucosal Scarring Index (MSI) [15], which evaluates five parameters (width, height/contour, color, suture marks, and overall appearance), with total scores ranging from 0 (no scar) to 10 (severe scarring).

Study settings

The study recruitment was from the outpatient dental clinics of the Oral Medicine and Periodontology Department in the Faculty of Dentistry of Cairo University and MSA University. Recruitment began in November 2023, and all surgical procedures were completed by September 2024. The final follow-up and data collection were completed in January 2025. The surgical procedures were performed in the postgraduate periodontology clinics in the Faculty of Dentistry of Cairo University and the periodontology clinics in the Faculty of Dentistry of MSA University.

Inclusion and exclusion criteria

The study included systemically healthy individuals aged between 18 and 40 years who exhibited an aberrant mandibular labial frenum with inadequate keratinized gingiva. Eligible participants were required to have a shallow vestibular sulcus, a thin gingival phenotype, and good oral hygiene, demonstrated by a gingival and plaque index of less than 20%.

Participants were excluded if they were smokers or had any physical disabilities that could interfere with maintaining proper oral hygiene. Individuals taking medications known to impair wound healing, or those diagnosed with systemic conditions that could delay tissue repair, were also excluded. Additional exclusion criteria included the presence of a deep vestibular sulcus, thick gingival phenotype, or a history of previous frenular surgeries. Furthermore, individuals who had undergone any form of periodontal surgery within the six months preceding the trial or who were pregnant or lactating at the time of recruitment were not considered eligible for participation.

Sample size

Twelve cases were agreed to be included in the study based on expert opinion (10 cases with two dropouts). 

Pre-surgical protocol

All patients underwent a comprehensive preoperative evaluation that included medical, clinical, and radiographic assessments.

From the medical standpoint, a detailed history was obtained to exclude systemic conditions or medications known to impair wound healing, such as uncontrolled diabetes, immunosuppression, bleeding disorders, or anticoagulant therapy. Vital signs, including blood pressure, pulse, and temperature, were recorded, and female patients were screened for pregnancy or lactation.

Clinical assessment included full-mouth periodontal charting, with plaque and gingival indices recorded for each patient. Only individuals with a plaque score below 20% were considered eligible for surgery. The gingival phenotype was determined visually and by probe transparency and classified as thin or thick, while vestibular depth was noted as either shallow or adequate. The width of keratinized tissue (WKT) at the mandibular midline was measured as the distance from the gingival margin to the mucogingival junction using a calibrated periodontal probe. 

The frenum was assessed through inspection and functional testing. Its insertion was classified according to the system of Mirko et al. into mucosal, gingival, papillary, or papilla-penetrating types [3]. A pull or blanching test was then performed by retracting the lip to evaluate the movement of the marginal gingiva and any color changes along the gingival margin. A frenum was diagnosed as aberrant if it demonstrated coronal insertion close to the gingival margin or papilla, produced blanching or mobility of the marginal gingiva on traction, or caused functional limitations such as a shallow vestibular depth or compromised oral hygiene. Standardized intraoral photographs were taken to document baseline findings.

Radiographic evaluation consisted of periapical radiographs of the mandibular anterior region to exclude bone pathology and to establish baseline alveolar bone levels. Following these assessments, all patients received professional mechanical plaque removal (PMPR) together with individualized oral hygiene instructions. Compliance was reassessed after six weeks, and only those who maintained plaque scores below 20% were scheduled for surgical intervention.

Patients were thoroughly informed about the nature of the procedure, potential risks and benefits, alternative treatment options, and the expected outcomes. Two copies of written informed consent in Arabic language were signed by both the patient and the operator, with each party keeping a copy of the form.

Surgical protocol

Frenotomy Procedure

**Figure 1:**
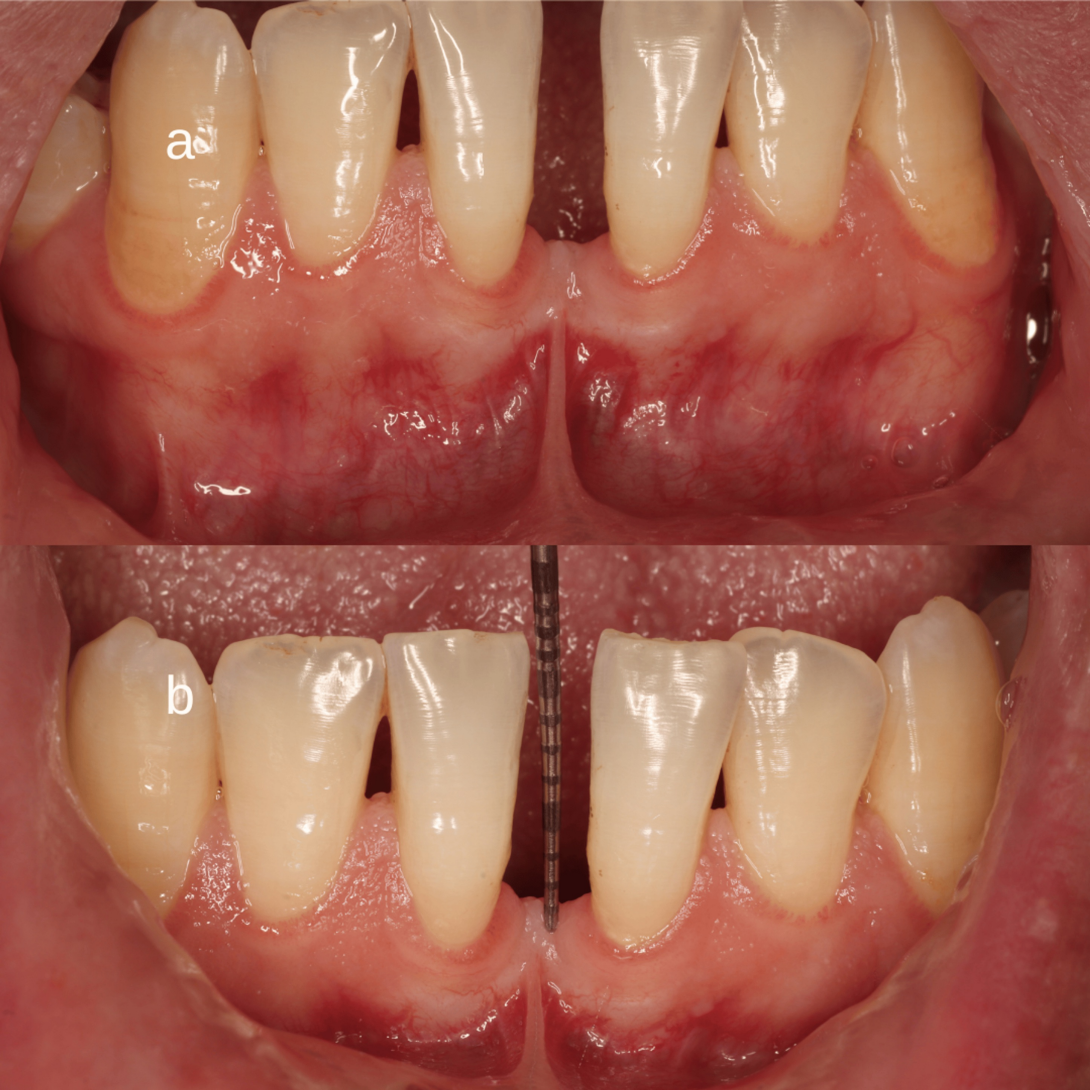
Preoperative photograph showing an aberrant mandibular labial frenum. a) Blanching of the gingival margin was noted while performing blanching test. b) Distance from the incisal edge to the frenum's tip was recorded using a periodontal probe to record the amount of relapse later on.

The area of the aberrant frenum (area of interest) was confirmed using the blanching test (Figure 1). This procedure was done by strictly stretching the lip until blanching or mobility of the free gingival margin was observed. As the study was a one-arm case series trial, blinding of the examiner at the beginning of the surgical procedure was not possible due to the nature of the study and intervention.

All patients underwent a standardized frenotomy procedure and were asked to rinse with chlorhexidine gluconate mouthwash 0.12% for one minute. Local infiltration was done using a local anaesthetic solution. After achieving adequate anaesthesia, the frenum was isolated using gauze and tension applied with traction.

**Figure 2:**
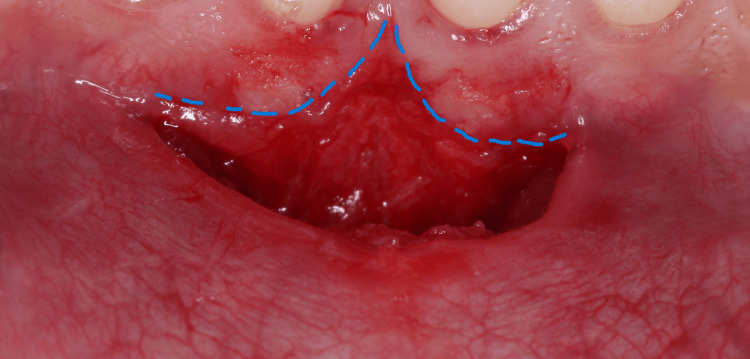
Frenotomy procedure C-shaped split‑thickness incisions are made on both sides of the frenum, followed by blunt dissection to apically reposition the frenum.

**Figure 3:**
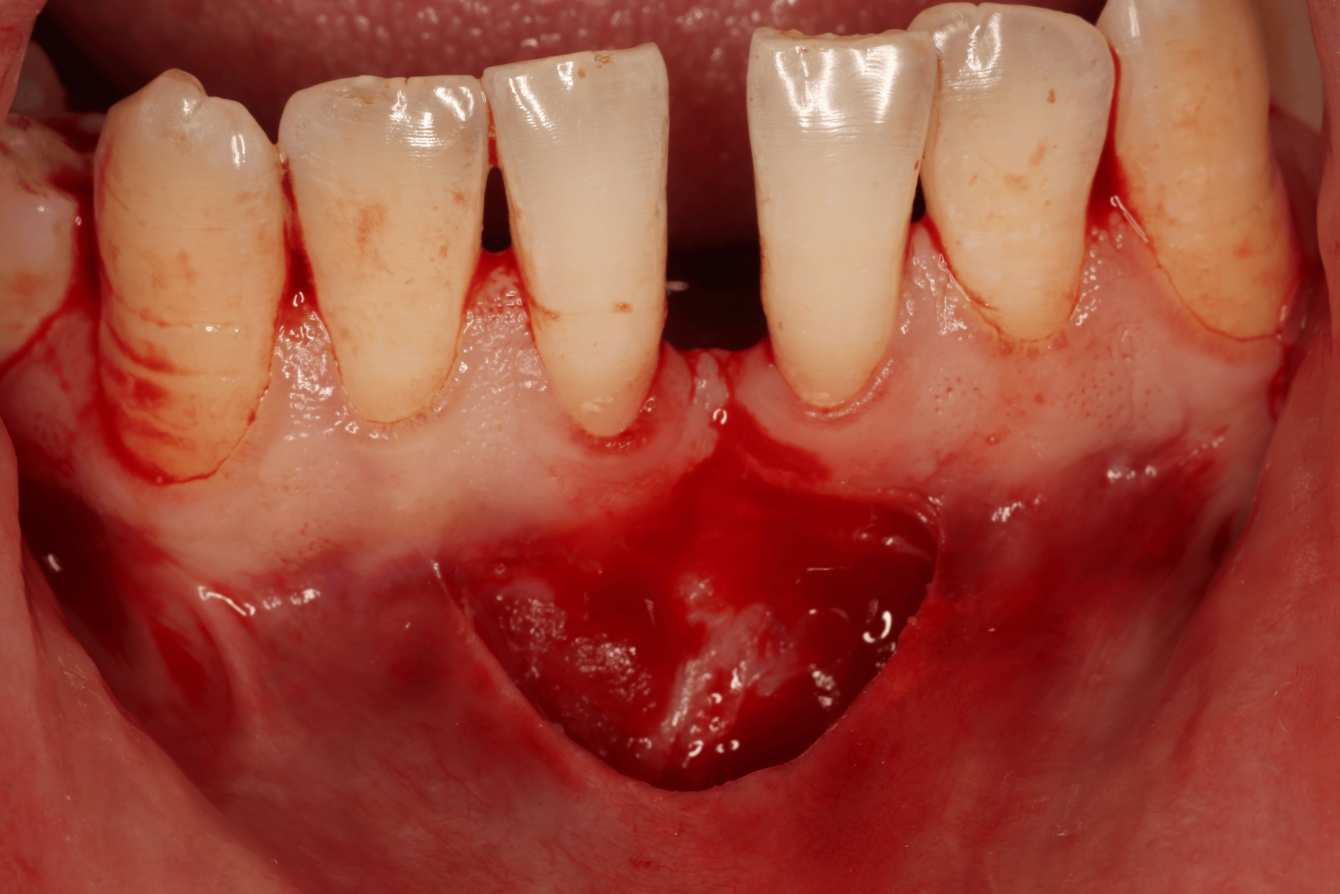
Free gingival graft recipient site preparation Apical displacement of muscle fibers following frenotomy to create an adequate recipient bed for free gingival graft placement

A C-shaped or oblique split-thickness incision was placed on the two sides of the frenum using a 15c blade with respect to tooth 41, moving to the tip of the frenum and then sliding down to the mucogingival junction opposite to tooth 31 (Figure 2), which was then followed by blunt dissection to separate the underlying muscle fibers from the periosteum relocating the frenum to a more apical level using mucoperiosteal elevator (Figure 3).

**Figure 4:**
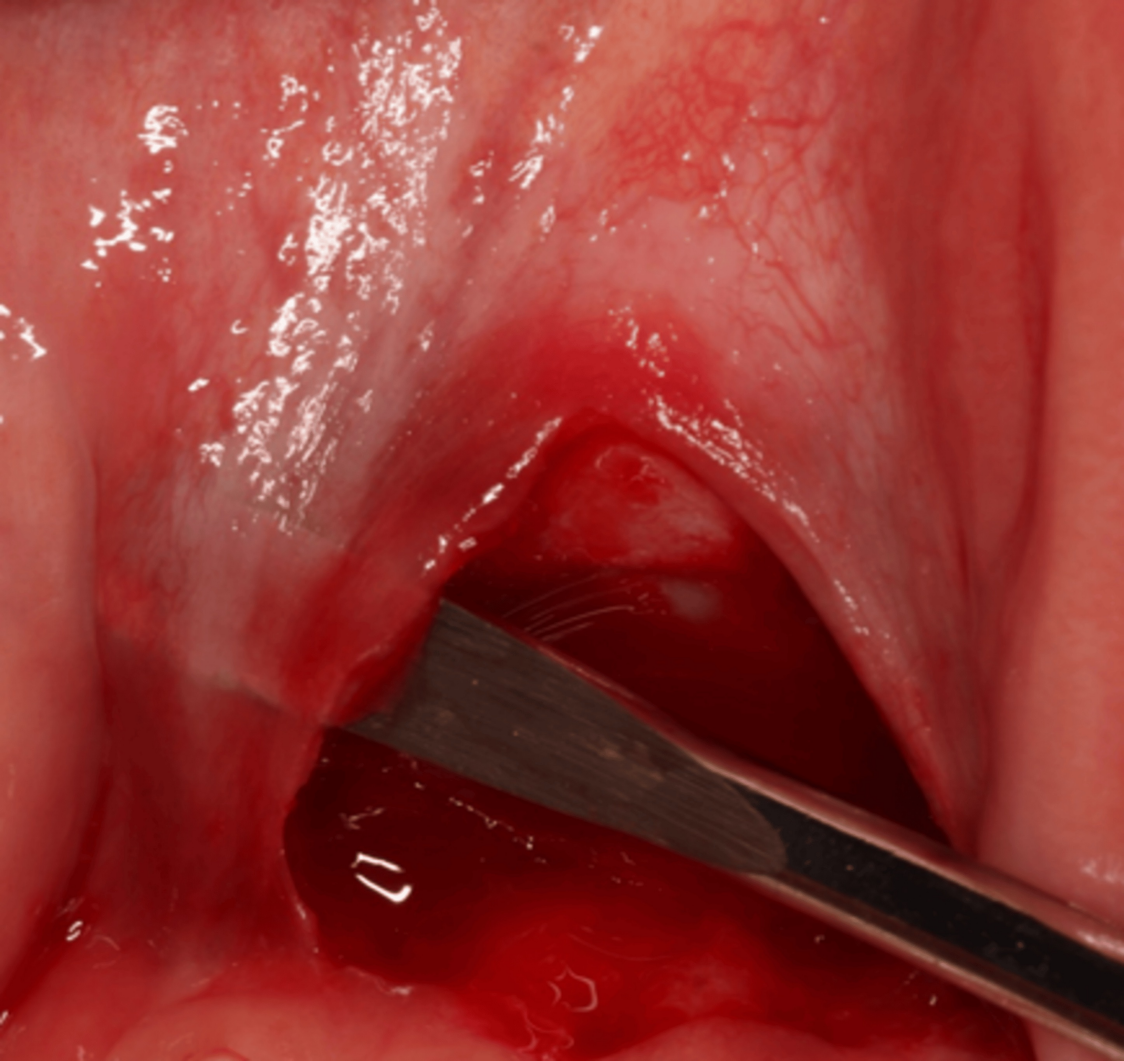
Undermining of flap edges following frenotomy Undermining of the flap margins was performed using a mucoperiosteal elevator to separate the epithelium from the underlying lip mucosa.

Undermining of the edges of the flap was done using a mucoperiosteal elevator to separate the epithelium from the underlying lip mucosa (Figure 4).

Haemostasis was achieved by a gauze compression moistened with saline and placed over the recipient bed until graft placement. Care was taken to preserve the vestibular depth and avoid unnecessary trauma to adjacent tissues.

FGG Harvesting

**Figure 5:**
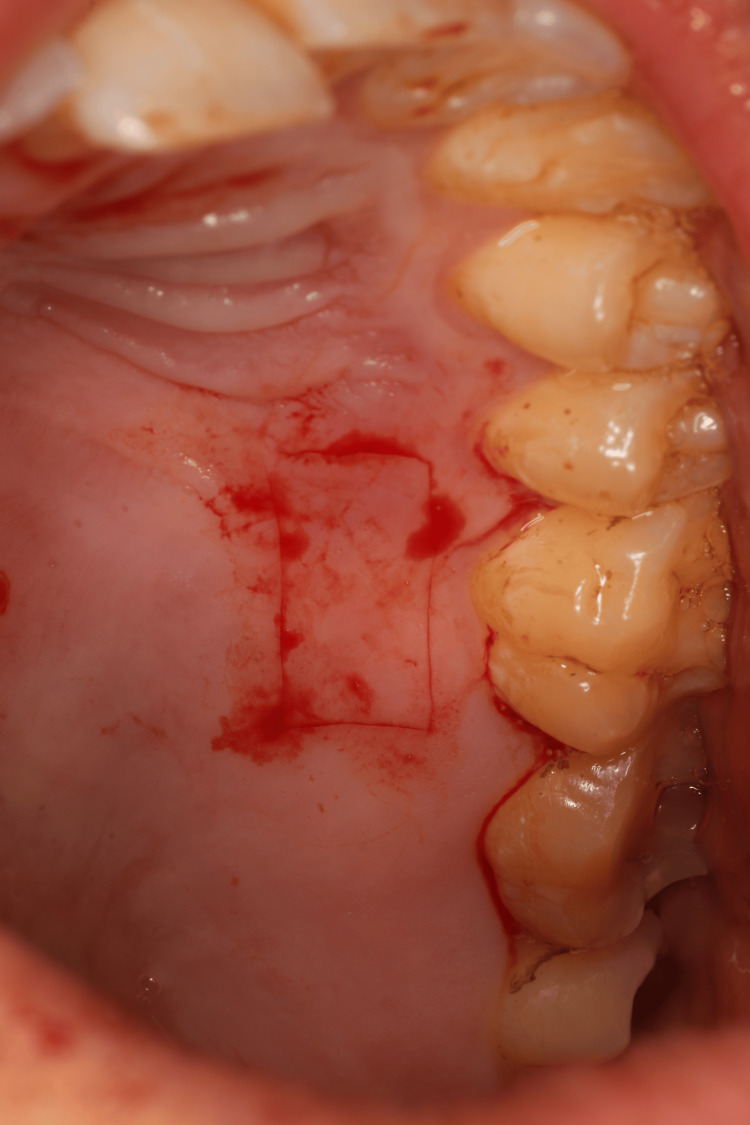
Outline of the graft marked with a periodontal probe Usually 10–15 mm in length and 5–7 mm in width

The donor tissue was harvested from the palatal mucosa, typically in the premolar to first molar region, where keratinized tissue was sufficient. The donor site was anesthetized using local infiltration of 2% lidocaine with 1:100,000 epinephrine. The outline of the graft was marked with a periodontal probe and a sterile tin foil template to achieve a dimension appropriate for the recipient site (usually 10-15 mm in length and 5-7 mm in width) (Figure 5) [16]. 

**Figure 6:**
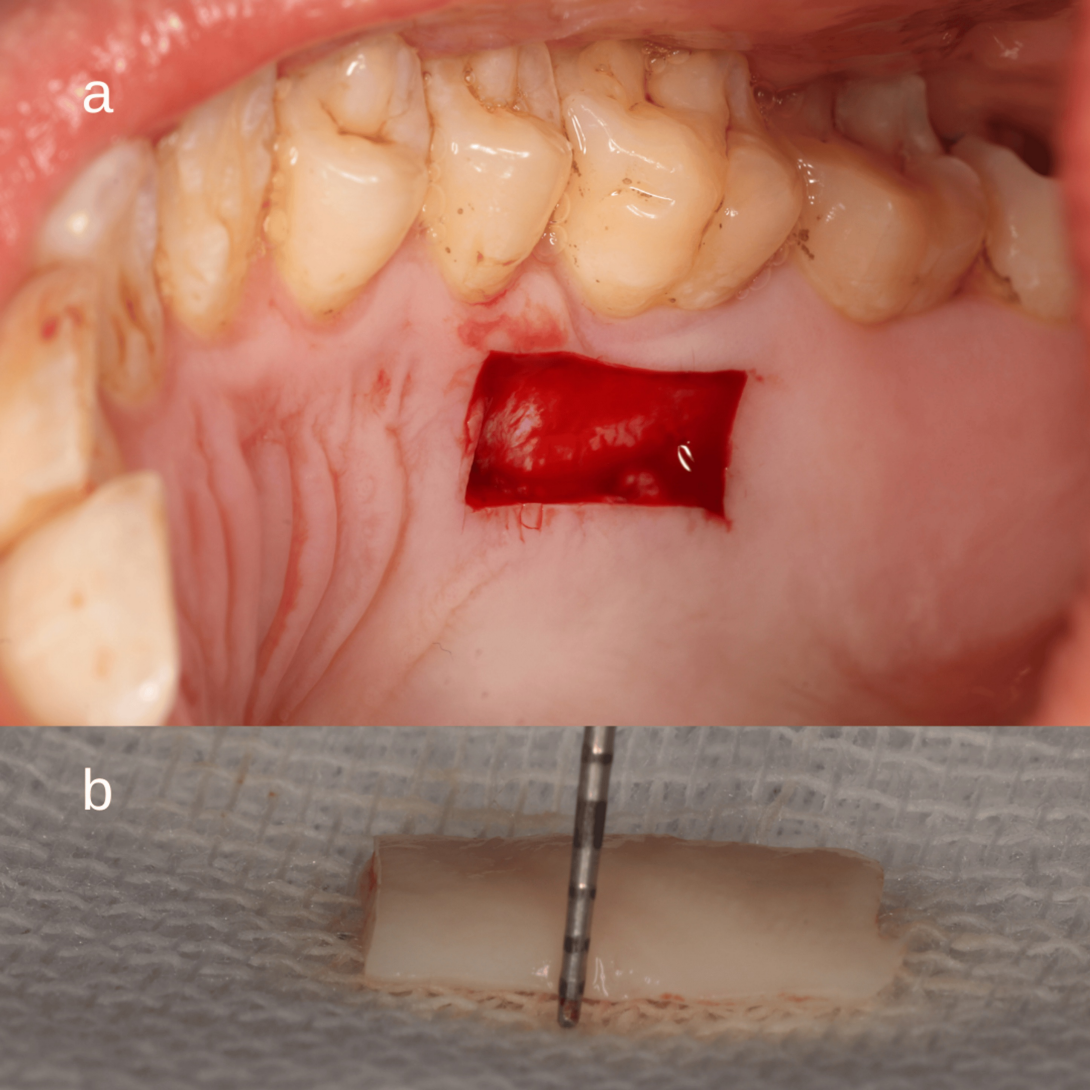
Free gingival graft harvested from the palate a) Palatal donor site after harvesting. b) Graft thickness was approximately 1 mm thick.

A shallow partial-thickness incision was made using a No. 15 blade to elevate a graft approximately 1-1.5 mm thick, ensuring the removal of the epithelium and part of the underlying connective tissue without involving the periosteum (Figure 6). The graft was immediately transferred to a sterile saline-moistened gauze to maintain hydration. Haemostasis at the donor site was achieved using gauze compression and application of a haemostatic agent if needed.

**Figure 7:**
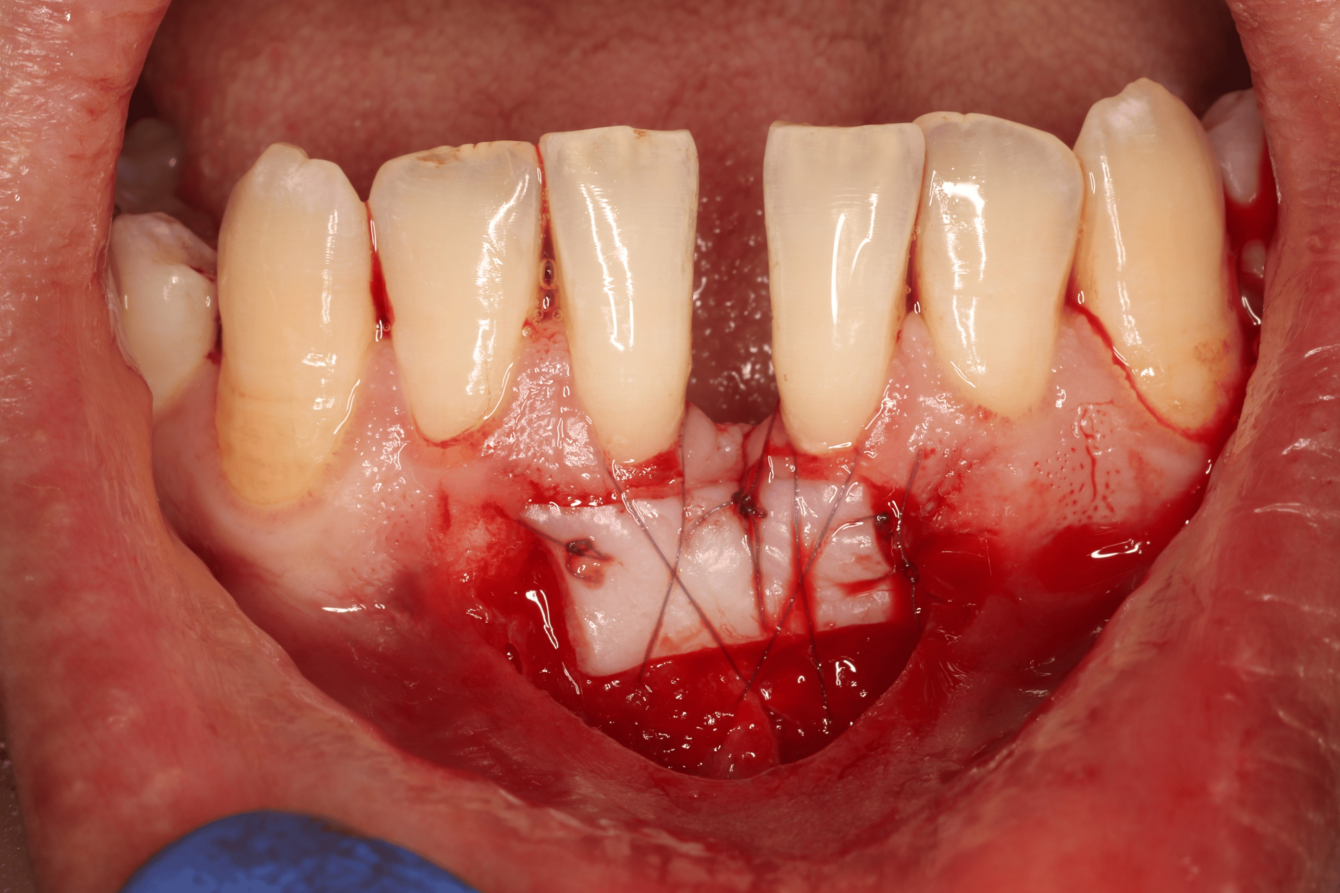
Free gingival autograft stabilized using 6-0 Vicryl sutures Free gingival graft stabilized at the edges with interrupted sutures, followed by two cross‑mattress sutures for additional stabilization and adaptation for the graft.

The recipient site was prepared by de-epithelializing the adjacent mucosa to expose a suitable connective tissue bed, ensuring optimal graft adaptation. The harvested FGG was then trimmed as needed and positioned at the recipient site with the epithelial side facing outward. The graft was secured using 6-0 resorbable braided sutures, employing interrupted and cross-mattress sutures to ensure passive adaptation without tension or folding (Figure 7).

Light pressure, using gauze soaked in a saline solution, was applied for two to three minutes to aid in hemostasis and promote intimate contact between the graft and recipient bed, limiting the dead space. Postoperative instructions were given, including analgesics and antimicrobial rinses, and follow-up visits were scheduled to monitor healing and remove sutures after seven to 10 days.

Post-surgical protocol

All patients received the same postoperative instructions, medications, and follow‑up protocol. Patients were advised to avoid mechanical trauma to the surgical sites and refrain from brushing or flossing the area for at least two days. A soft diet was recommended for five to seven days, and patients were instructed not to disturb the surgical sutures. At two to three days postoperatively, patients were instructed to resume gentle tooth brushing with a soft-bristle brush away from the surgical site.

Ibuprofen 600 mg was prescribed for pain management whenever needed only, and an antiseptic 0.12% chlorhexidine mouthwash was prescribed twice daily. Rinsing started 24 hours post-surgery and continued for two weeks. Professional prophylaxis was performed monthly during the first three months post-surgery to maintain a plaque-free environment.

Recall Appointments (T1, T2, T3, T4, and T5)

T1: On the fifth postoperative day, the patients were recalled to the dental clinic to record their mucosal healing using the IPR wound healing index (Inflammatory phase).

T2: On the seventh postoperative day, pain scores were collected on an NRS index score.

**Figure 8:**
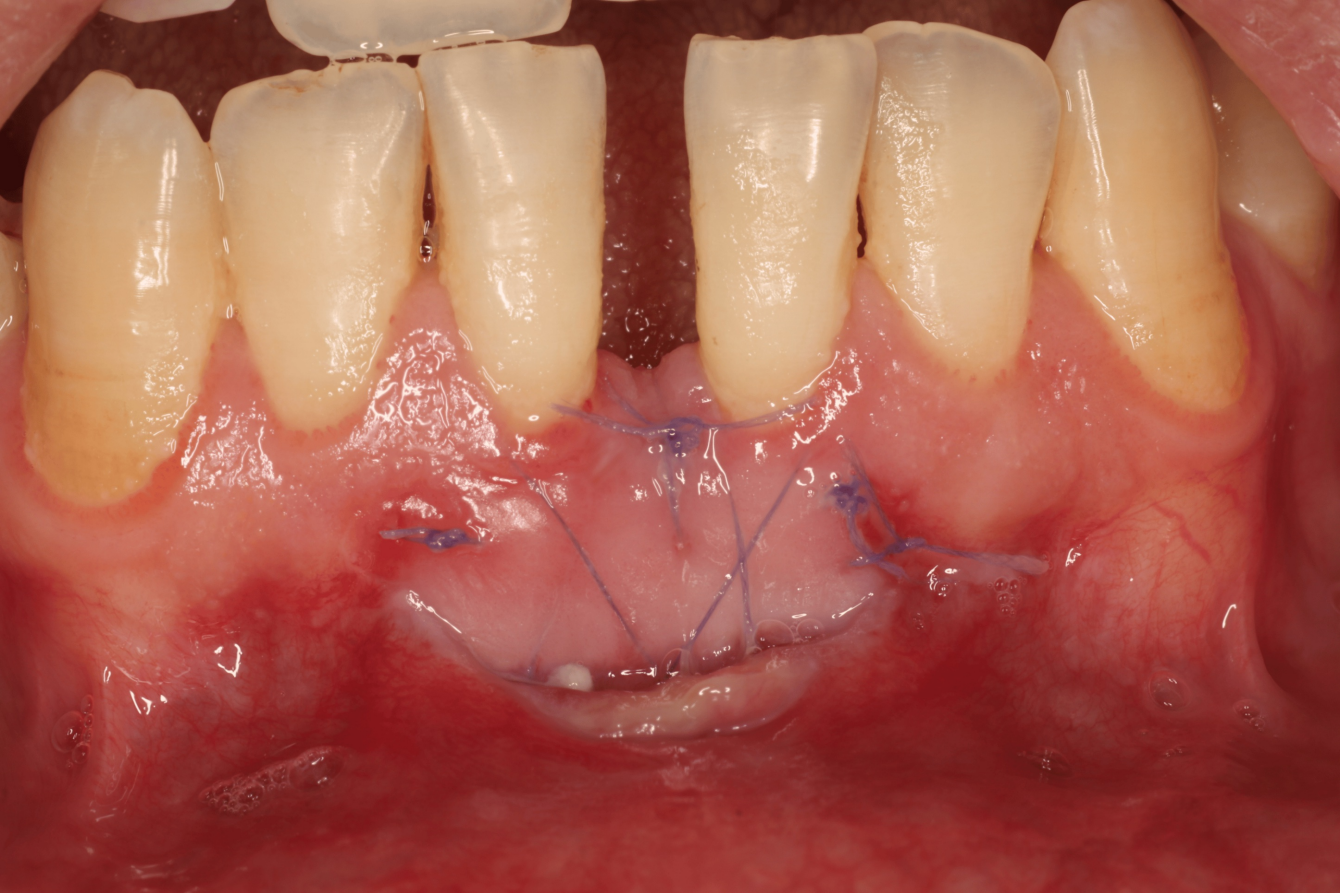
Postoperative evaluation after two weeks At 14 days, partial re-epithelialization and whiter tissue color were observed (scores 0 each). The scar was less than 2 mm with some contour regularity (score 1). No suppuration was present (score 1), and the patient reported no pain (VAS 0, score 1), giving a total score of 3/5 for the proliferative phase.

T3: On the 14th postoperative day, the patients were recalled to the dental clinic to record their mucosal healing using the IPR wound healing index (proliferative phase)(Figure 8).

**Figure 9:**
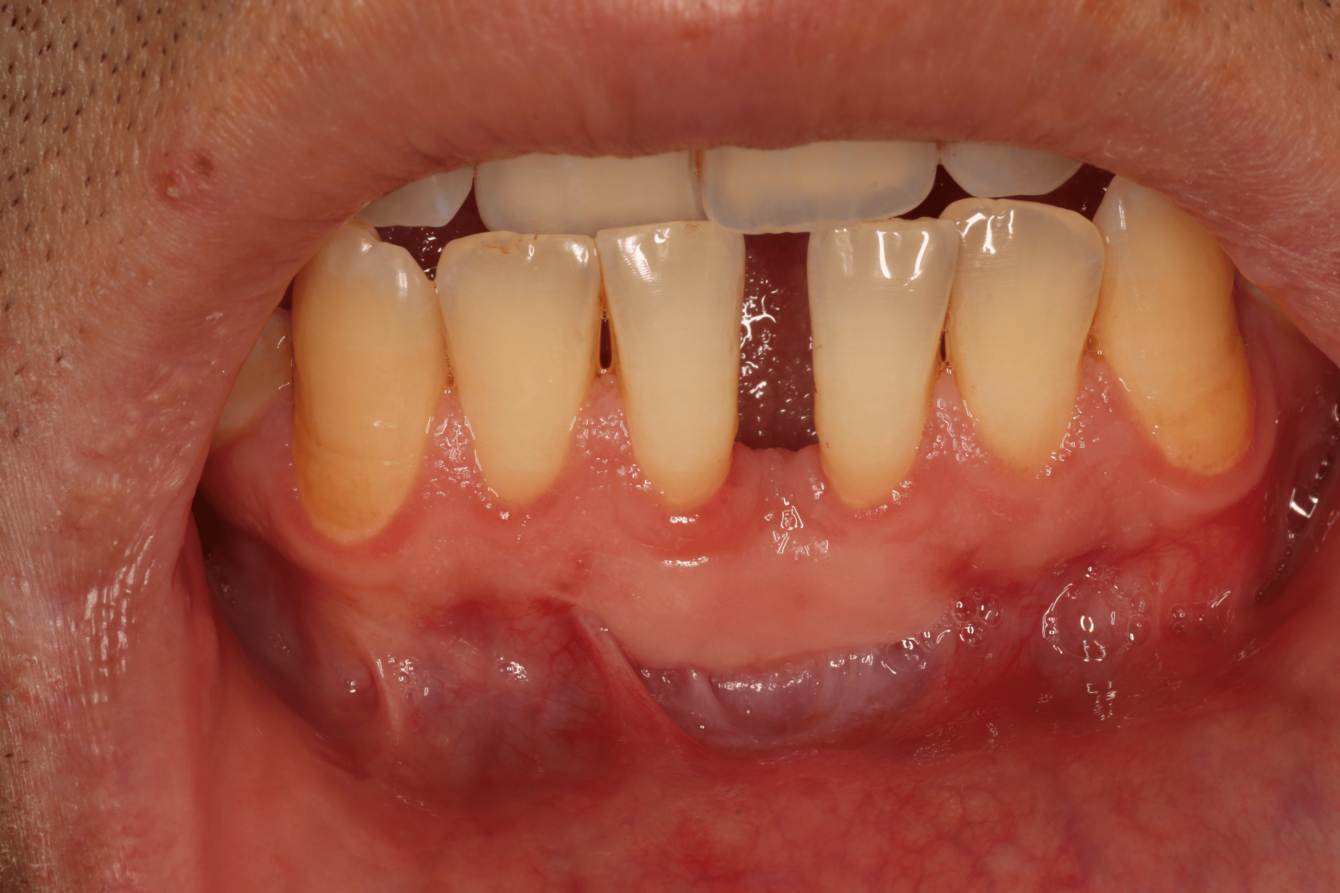
Postoperative evaluation after six weeks At six weeks, the scar was less than 2 mm wide with improved contour regularity, warranting a score of 1. The tissue color remained slightly whiter than the adjacent gingiva (score 0), and the patient reported no pain (VAS 0), contributing an additional point. This resulted in a total score of 2 out of 3 for the remodeling phase.

T4: After six weeks postoperative, the patients were recalled to the dental clinic to record their mucosal healing using the IPR wound healing index (remodelling phase) (Figure 9).

**Figure 10:**
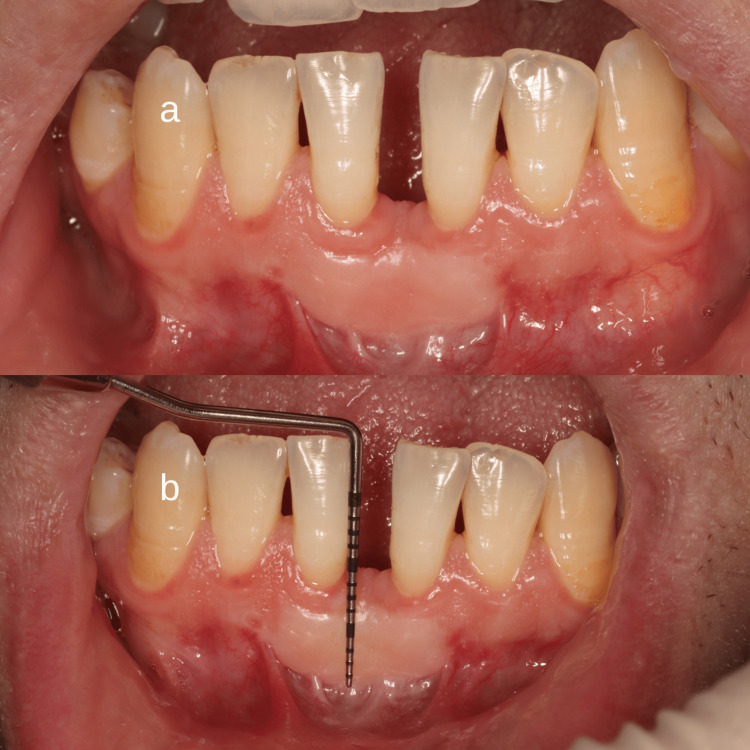
Postoperative evaluation at three months a) Three‑month follow‑up showing successful healing. Mild‑to‑moderate scar formation (MSI score: 4) is visible without functional limitation. Keratinized tissue width increased from 2 mm to 10 mm (gain: 8 mm). b) Relapse assessment, measured from the incisal edge of the mandibular central incisor to the tip of the frenum, showed that the postoperative distance was maintained with no reduction over the three‑month follow‑up.

T5: After three months postoperatively, the patients were recalled to the dental clinic to record the gain of keratinized tissue width, the scar tissue formed using MSI index score, and to measure the amount of frenum relapse using a periodontal probe (Figure 10).

Data analysis and statistical method

The mean and standard deviation values were calculated for each test. Data were explored for normality using Kolmogorov-Smirnov and Shapiro-Wilk tests. WKT and relapse data showed parametric (normal) distribution, while pain, mucosal healing, and scar index showed non-parametric (not-normal) distribution.

For data with a normal distribution (parametric data), repeated measures ANOVA was used to evaluate changes across multiple time points within the same individuals, followed by a t-test. For non-normally distributed data (non-parametric data), the Friedman test was used to assess changes over time, followed by Wilcoxon signed-rank tests for pairwise comparisons.

The significance level was set at P < 0.05. Statistical analysis was performed with IBM SPSS Statistics for Windows, Version 25.0 (IBM Corp., Armonk, NY).

## Results

The study included 12 patients (three males, nine females) with an age range of 21-38 years with diagnosed aberrant frenum attachment; the mean age was 28.83 ± 6.49 years. All 12 patients completed follow‑up with no loss to follow‑up. After surgical intervention and during a three-month follow-up period, healing was uneventful. After three months, there was minimal scar formation with a scar index of 4.17 ± 0.94 (MSI, 0-10).

Primary outcome

Changes in the WKT

There was a significant gain in the WKT after frenectomy and FGG detected after two weeks, two months, and three months (p < 0.001) after surgical intervention. The maximum gain in WKT was detected at three months (6.8 mm ± 0.44 mm) compared to baseline (Table 1).

**Table 1:** Changes in the width of keratinized tissue (WKT) over time. WKT: width of keratinized tissue, SD: standard deviation, p: probability value, F-value: test statistic from repeated-measures ANOVA Superscript letters (a, b, c) indicate statistical grouping: values with different letters are significantly different at p < 0.05, while values sharing the same letter are not significantly different.

Variables	WKT (mm)		
	Mean		SD
Baseline	2.083^ c^		0.336
After two weeks	7.333^ b^		0.62
After two months	8.000^ ab^		0.55
After three months	8.833^ a^		0.534
p-value	<0.001*		
F-value	77.173		
Gain	6.833	0.441	

Secondary outcome

Pain

**Figure 11:**
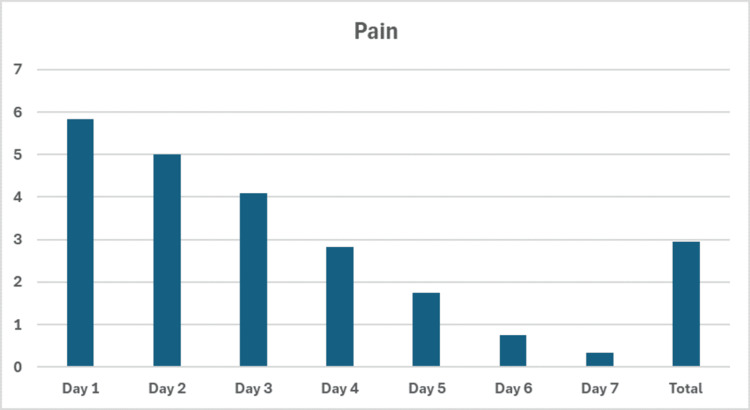
Chart representing pain scores. NRS: Numeric Rating Scale, ranging from 0 (“no pain”) to 10 (“worst pain imaginable”). Values represent mean ± standard deviation (SD). p = probability value calculated with non-parametric test (Friedman).

The pain perceived by the patients reached its maximum at day 1 (5.8 ± 0.34, NRS, 0-10) and was significantly reduced by day 7 (0.33 ± 0.14, NRS, 0-10) (p = 0.002). There was a significant reduction in pain scores from day 2 up to day 7 (Figure 11).

IPR Wound Healing Score (Mucosal Healing)

The inflammatory phase of the IPR wound healing score was assessed after five days and reached 5.33 ± 0.25 (IPR "inflammatory phase", range 0-8), denoting successful healing (Table 2). 

**Table 2:** Mean and standard deviation (SD) values of mucosal healing IPR: Inflammatory-Proliferative-Remodeling wound healing index, SD: standard deviation. Phases: Inflammatory (day 5), Proliferative (day 14), and Remodelling (six weeks).

Variables	Mean	SD	Median
Inflammatory	5.333	0.256	5.5
Proliferative	4	0.213	4
Remodeling	2.167	0.112	2
Total score	11.5	0.452	11.5

The proliferative phase evaluated after 14 days was 4.0 ± 0.23 (IPR "proliferative phase", range 0-5), also denoting successful healing (Table 2). 

The remodeling phase was (2.17±0.11) after six weeks (IPR "remodeling phase", range 0-8), which indicates successful healing. The total mean score of the IPR scale was 11.50 ± 0.45 (IPR, 0-16), which indicates excellent healing. In 83.3% of the treated cases, the IPR scores pointed to excellent healing, while acceptable healing was detected in 16.7% of the cases (Table 3). 

**Table 3:** Mucosal healing outcomes at six weeks following frenotomy with a free gingival graft IPR: Inflammatory-Proliferative-Remodeling wound healing index. p: probability value, χ²: chi-square value from Friedman test. : number of cases. *p < 0.05.

Variables		Mucosal healing			
		n	%	p-value	Chi-square
Total score	Acceptable healing (5-10)	2	16.70%	0.021*	5.333
(After six weeks)	Excellent healing (11-16)	10	83.30%		

Relapse

After three months of surgical intervention, the frenum was located at a more apical level than baseline by 4.3 mm ± 0.43 mm, with a significant difference between its baseline and three-month location (p < 0.001).

## Discussion

Aberrant frenal attachments, positioned near the gingival margin, can compromise gingival health by limiting plaque control, inducing recession, and increasing the risk of relapse after orthodontic treatment [4,17]. Although conventional frenectomy effectively addresses these issues [6], it is often associated with complications such as scarring, recurrence, and prolonged healing [2,5]. By contrast, frenotomy, a more conservative technique, offers reduced trauma and improved healing outcomes while preserving tissue integrity [7,8].

Combining frenotomy with FGGs provides both functional release and soft tissue augmentation, addressing mucogingival concerns like narrow keratinized tissue, shallow vestibules, and relapse [18]. FGG is the gold standard for increasing keratinized tissue width and reinforcing the surgical site against mechanical stress and muscular pull [9].

This study evaluated a dual approach of frenotomy and FGG in adults with aberrant labial mandibular frena, aiming to ensure long-term periodontal stability, improve mucosal healing, and reduce pain and scar formation. To our knowledge, this is the first study to present a detailed surgical protocol of frenotomy combined with FGG application.

Our study demonstrated a substantial and statistically significant gain in the WKT following the combined frenotomy and FGG approach, with a mean increase from 2.08 mm at baseline to 8.83 mm at three months postoperatively (p < 0.001). The FGG serves a dual purpose by not only increasing the protective keratinized zone but also resisting mechanical tension from high frenular attachments, thus preventing relapse.

These findings are in line with previous studies reporting similar gains that achieved up to 6.5 mm increase using titanium tack-fixed FGGs in mandibular regions [19], while another reported 6.2 mm using tension-free techniques and optimal suturing [10]. Compared to alternative methods, such as collagen matrices or living cellular constructs, FGG consistently yielded superior outcomes in WKT gain [20], although the alternatives may offer esthetic advantages. Notably, the early stabilization observed between the second- and third-month marks aligns with prior reports, indicating that most dimensional changes occur within the first six to eight weeks after graft placement [21].

Despite involving two surgical sites, pain scores showed a rapid and statistically significant decline from a mean of 5.83 on day 1 to 0.33 by day 7, reflecting the conservative and atraumatic nature of the procedure (p < 0.001). These results suggest that frenotomy, as a less invasive alternative to full frenectomy, paired with meticulous donor site management, can significantly reduce early postoperative discomfort.

This pattern is comparable to other studies using scalpel-based methods that observed similar day 1 pain scores with rapid recovery by day 7 [22]. Although laser-assisted frenotomies have shown slightly lower early pain scores [23], they often come with slower healing due to tissue carbonization. Moreover, autogenous graft-involving procedures typically cause more discomfort than collagen-based substitutes, yet our study's results indicate that careful surgical handling and patient management can mitigate this pain effectively [24].

Wound healing was evaluated using the IPR scale, and the mean total score of 11.5 out of 16 classified most patients (83.3%) in the “excellent” category. The phase-specific scores, i.e., 5.33 (inflammatory), 4.0 (proliferative), and 2.17 (remodeling), reflected progressive and successful healing. This outcome supports the surgical technique’s effectiveness in promoting rapid epithelialization, revascularization, and tissue maturation.

These findings align with the work of Burkhardt, who emphasized microsurgical principles and tension-free graft adaptation as critical for optimal healing [25]. Proper graft stabilization, maintained through careful split-thickness bed preparation and passive suturing, and good adaptation on the recipient site is likely to contribute to the favorable scores. In addition, classical insights from Sullivan and Atkins (1968) and Allen (1994) regarding the importance of graft-host contact are echoed in these outcomes.

Relapse was assessed by measuring the distance between the incisal edge and the frenum insertion, showing a mean of 4.33 mm apical migration (from 11.58 mm to 15.92 mm, p < 0.001). These findings confirm the mechanical and biological efficacy of combining frenotomy with FGG in preventing reattachment or regrowth of the frenum, verifying the stability of the outcomes.

This result is supported by another study that used a similar measurement method and found significant improvements in frenal position following release procedures [26]. Historical evidence suggests higher relapse rates when soft tissue augmentation is omitted [2]. The FGG in our study likely provided a barrier against the muscle reattachment and enhanced tissue resistance [9]. By contrast, procedures lacking graft reinforcement, including some laser-based methods, have reported partial or complete relapse [27].

Scar formation was minimal, with a mean MSI score of 4.17 ± 0.94, suggesting mild to moderate scarring that is considered clinically acceptable in the mandibular anterior region. The conservative design of frenotomy, along with passive adaptation of the FGG, likely minimized excessive tissue trauma and tension, two primary drivers of fibrotic scarring.

These findings mirror previous reports that emphasized how surgical tension and wide incisions can lead to hypertrophic or fibrotic healing [28,29]. Tension-relieving suture techniques and preservation of anatomical continuity appear to have played a significant role in controlling scar formation. Compared to classical frenectomy techniques, which often heal by secondary intention and show higher scar scores [2], our approach appears to offer superior aesthetic and functional results.

The favorable outcomes observed with the frenotomy-FGG approach are most likely explained by two complementary mechanisms: mechanical displacement and reduced surgical trauma. Mechanically, apical relocation of the frenal fibers combined with placement of an FGG creates a stable, keratinized soft-tissue barrier that resists coronal reattachment of the frenum and improves long-term periodontal stability, an effect described previously in clinical reports of graft-assisted frenal surgery [30].

Second, because frenotomy repositions the frenum rather than removing it entirely, the procedure is less destructive to soft tissues and their blood supply; preservation of tissue continuity and vascularization likely contributed to the modest postoperative pain and the rapid, favorable wound healing observed in our study. This principle - that less invasive mucogingival approaches preserve vascularity and reduce trauma, improving early healing - has been noted in reviews comparing more conservative mucogingival techniques to more extensive excisional procedures [5].

The study’s main limitations include a small sample size (12 participants), a short follow-up period (three months), and the lack of a control group. Moreover, there is a lack of possibility for blinding of the clinician and outcome assessor due to the nature of the study and the intervention. These factors limit the statistical strength and generalizability of the findings. In addition, some outcomes, like pain and scarring, were subjective, potentially influenced by bias. Operator variability and the absence of radiographic or histological evaluations further weaken the objectivity and reproducibility of the results. Future research should include larger, randomized controlled trials with longer follow-up periods (6-12 months) and control groups for comparison. Objective assessment methods like imaging and histology, as well as standardized scoring for healing and scarring, are advised. Studies should also evaluate patient-centered outcomes (comfort, esthetics, and oral hygiene impact) and consider cost-effectiveness. Exploring enhancements like microsurgical or laser-assisted techniques may further improve outcomes and reduce morbidity.

## Conclusions

This clinical case series evaluated a combined surgical technique - frenotomy with FGGs - for treating aberrant mandibular labial frenum in adults. The method aimed to address issues like insufficient keratinized tissue, shallow vestibule, frenum recurrence, and risk of gingival recession. The outcomes of the study suggest that the combined approach can enhance peri-implant and periodontal stability in cases where soft tissue inadequacy compromises long-term success.
